# Relationship between Procedural Tactical Knowledge and Specific Motor Skills in Young Soccer Players

**DOI:** 10.3390/sports4040052

**Published:** 2016-11-17

**Authors:** Rodrigo Aquino, Renato Francisco R. Marques, Grégory Hallé Petiot, Luiz Guilherme C. Gonçalves, Camila Moraes, Paulo Roberto P. Santiago, Enrico Fuini Puggina

**Affiliations:** 1 CIFI2D, Faculty of Sport, University of Porto, Porto 4200-450, Portugal; ghpetiot@icloud.com; 2 Medicine School of Ribeirão Preto, University of São Paulo, Ribeirão Preto 14049-900, Brazil; paulosantiago@usp.br (P.R.P.S.); enrico@usp.br (E.F.P.); 3 School of Physical Education and Sport of Ribeirão Preto, University of São Paulo, Ribeirão Preto 14040-030, Brazil; renatomarques@usp.br (R.F.R.M.); camimoraes@usp.br (C.M.); 4 Department of Performance Analysis, Botafogo Football Club, Ribeirão Preto 14096-070, Brazil; luizgoncalves@usp.br

**Keywords:** technical skill, tactical procedural knowledge, offensive tactics, football, soccer

## Abstract

The purpose of this study was to investigate the association between offensive tactical knowledge and the soccer-specific motor skills performance. Fifteen participants were submitted to two evaluation tests, one to assess their technical and tactical analysis. The motor skills performance was measured through four tests of technical soccer skills: ball control, shooting, passing and dribbling. The tactical performance was based on a tactical assessment system called FUT-SAT (Analyses of Procedural Tactical Knowledge in Soccer). Afterwards, technical and tactical evaluation scores were ranked with and without the use of the cluster method. A positive, weak correlation was perceived in both analyses (rho = 0.39, not significant *p* = 0.14 (with cluster analysis); and rho = 0.35; not significant *p* = 0.20 (without cluster analysis)). We can conclude that there was a weak association between the technical and the offensive tactical knowledge. This shows the need to reflect on the use of such tests to assess technical skills in team sports since they do not take into account the variability and unpredictability of game actions and disregard the inherent needs to assess such skill performance in the game.

## 1. Introduction

The structure of team sports obliges the execution of open tasks due to a high variability and unpredictability. As defined by Thorpe, Bunker and Almond [1], invasion games can be recognized by a direct and continuous interaction between teammates, opponents, free space and ball in the same space, which provides unpredictable results [2]. However, a player must know what to do and how to do it in order to determine his actions in the course of the game. We refer to this knowledge as the tactical knowledge.

We understand tactical knowledge as “*knowledge of*” general solutions that can be applied to solve game situations [3]. These solutions apply to situations of simple opposition to complex configurations of play [4]. That knowledge is to be retrieved according to the context and the player’s ability to recognize what he has perceived. Since the use of that knowledge can be observed through the execution of tactical actions in the play, assessment tools in team sports measure the players’ knowledge through their performance in realizing actions while in play [5].

In contrast, we define motor skills in soccer as the set of abilities needed to realize the body movements, i.e., technical mastery of the ball. These body movements would include both general sets of coordination movements allowing the player to dislocate on the field, as well as a modality-specific set of movements allowing the player to pursue the game objectives, such as passing or shooting the ball. The assessment of motor actions is generally realized observing isolated tasks (e.g., ball control, shooting, passing and dribbling). The assessed actions are consequently analyzed outside of the game context.

Nevertheless, action in sports is realized based on what the players know, although such knowledge is composed of “knowing how to do something” and “knowing what to do” [6]. This distinction is important first because the player has to deal with the particularity of the unrepeated situations, and second because even knowing what to do will not guarantee that he will execute it efficiently. In such conditions, the player must constantly realize tactical and technical actions both according to the context and his capabilities.

Assuming that the two sets of knowledge are intimately related through the execution of an action in the game, it is relevant to verify the relation between technical and tactical skills. Recent study published by Praça et al. [7] evaluated 24 federated soccer players with relatively high training experience and found a low correlation between the technical execution of actions measured outside of game and tactical knowledge. However, the authors did not assess young soccer players with relatively low training experience. In addition to maturation development, studies enlighten significant differences of technical and tactical performance between age levels, possibly due to the experience level of soccer training [8].

This study aims to identify possible associations between tactical knowledge and soccer-specific motor skills in soccer players evolving in the Under-11 age level, with validated and reproducible scientific protocols. However, we also hypothesize there is no correlation between tactical knowledge and motor skills for three main reasons: (i) the soccer-specific motor skills tests applied are performed outside of game conditions, i.e., the variability that obliges the players to explore and create opportunities for actions; (ii) the way soccer-specific motor skills are widely developed making young players repeat movements with the ball in a controlled environment; (iii) most of the tactical actions are realized without the ball, hence, good tactical actions from teammates provide good opportunities for the player in possession of the ball to pursue the team objective efficiently. 

## 2. Materials and Methods

### 2.1. Participants

The study was realized with 15 Under-11 soccer players affiliated with the academy of a professional club in the state of São Paulo, Brazil. The players had to have participated in training activities at the club for at least one year in order to minimize possible differences of technical and tactical knowledge between the participants. All players were competing in the top local league and they trained for approximately 80 min per training session, three days per week. The characteristics of the sample are described in Table 1. This study complies with the Code of Ethics of the World Medical Association (approved by the ethics advisory board of Swansea University); it was approved by the Research Ethics Committee of the Faculty of Philosophy, Sciences and Literature of Ribeirão Preto—USP (No. 133.230); and it was conducted in accordance with the Declaration of Helsinki. All legal tutors of the participants signed a consent form stating the procedures and aims of this study.

### 2.2. Procedures

This study was carried out over a two-week post-season training period in August of 2014. The players who participated were not involved in any other training or matches. On the first training day (Monday), anthropometric measures (i.e., height, body mass, % fat and maturity offset) and experience level (i.e., years of participation in regional and state leagues) were obtained. On the second day (Wednesday), the System of Tactical Assessment in Soccer (FUT-SAT) was applied to assess the procedural tactical knowledge. Finally, on the third day (Friday), players completed the soccer-specific motor skills assessment, including ball control, shooting, passing and dribbling tests. All measures were collected after applying the protocols to a natural grass field and by following the usual training schedule of the team (05:00 PM; temperatures from 27 °C (80.6 °F) to 33 °C (91.4 °F), without rain or wind). After one week, the participants were submitted to the same battery of assessments for test-retest reliability (Correlation matrix: range 0.91–0.99; Intra class coefficients: range 0.95–0.99). 

### 2.3. Instruments

#### 2.3.1. Analyses of Procedural Tactical Knowledge in Soccer (FUT-SAT)

The data related to tactical behavior of the participants was obtained based on the guidelines of the Tactical Assessment System for Soccer (FUT-SAT), validated by da Costa, Garganta, Greco, et al. [5]. The configuration of the test configures goalkeeper + three players versus three players + goalkeeper (GR3 + 3GR). The configuration is maintained in a game of four minutes in a soccer field of 36 m long by 27 m wide. A familiarization period of 30 s was pursued previous to the formal analysis period. The official rules of the game of soccer were applied with the exception of the offside rule.

The FUT-SAT assesses the tactical actions performed by the players according to the place of action on the field and according to ten fundamental principles of the game. These principles include five offensives (i.e., Penetration, Offensive Coverage, Depth Mobility, Width and Length, Offensive Unity) as well as five defensive principles (i.e., Delay, Defensive Coverage, Balance, Concentration, Defensive Unity). The tactical analysis was performed with video analysis software (SoccerAnalyser^®^).

According to the protocol proposed by Costa et al. [5], the tactical assessment consists of three procedures: (i) analyzing the tactical actions performed by the players, i.e., observing the completion of at least three consecutive contacts with the ball, the completion of a pass or the execution of a shot towards the opponent’s goal; (ii) evaluating and classifying these actions; and (iii) calculating the Index of Tactical Performance. For the purpose of this study, we have only considered the results obtained in the offensive phase (offensive index of tactical performance) because the soccer-specific motor skills tests performed with the ball can only be observed in offensive phases of the play. Consequently, the offensive index of tactical performance is obtained by calculating the average of the index of tactical performance of the five offensive tactical principles (i.e., Penetration, Offensive Coverage, Depth Mobility, Width and Length, Offensive Unity). We assumed that the higher the offensive index of tactical performance was, the higher the offensive procedural tactical knowledge would be (for more details see Costa. [5]). The participants were classified from highest to lowest according to the calculated index of tactical performance in order to obtain the final ranking.

#### 2.3.2. Soccer Specific Motor Skills Tests

All soccer-specific motor skills tests were previously validated for the assessment of soccer players [9,10] and have been extensively applied in previous studies [8,11,12]. 

Ball control [9]: The participants were oriented to remain positioned in a 9 m^2^ area delimited with cones. The objective of the test was to raise the ball with the feet, as many times as possible, without letting it touch the ground. The initial position of the ball had to be on the ground and the subject must not step out of the square. Two attempts were performed considering the best result amongst them.

Shooting [9]: The participants were positioned with the ball at 9 m away from the targets. The test consists of shooting the ball into one of the targets. The result was obtained according to the following scores: 1 point for shooting the ball in the central space, 2 points for the upper central space, 3 points for the side rectangles, and 5 points for the top square side. The target had the dimensions of 2 m high by 3 m wide. If the ball touched the material used to divide the spaces, the highest scoring was considered. Five attempts were performed and the best result was considered.

Passing [10]: The participants were instructed to perform the highest amount of passes to a target drawn on a wall for 20 s. The target was 1.22 m high by 2.44 m wide. The players were positioned in an area 1.83 m long by 4.23 m wide, at a distance of 1.83 m away from the target. Three attempts were performed in order to consider the best result. When the pass was off the target, one pass was deducted in the final count.

Dribbling [9]: According to the guidelines of the test, the players aimed to perform a route shaped like the letter “M” in the shortest time dribbling. The path was marked with cones within a space of 9 m × 9 m. If the participant would displace or flip any of the cones, he should stop and replace the cone to continue the test. Two attempts were realized, taking into account the fastest one.

For a better understanding of the operational structure of the motor skills tests, see the books on the related subject published by the *Portuguese Soccer Federation* [9] and Kirkendall et al. [10]. A second classification of the participants was made from the performance in these tests. The best performance resulted in first ranking and so on. A final classification was determined from the sum of the ranking in each test, so that the participant who had obtained the lowest sum was considered the best in technical ranking (Table 2). In case of a tie between participants both in tactical and in technical ranking, their ranking was determined according to the mean of their positions such as suggested by Pagano et al. [13].

We overcame the lack of consideration of the degree of difference between the test results performing a cluster analysis. We used the k-means method [14,15] and the algorithm powered by MATLAB^®^ (MathWorks, Natick, MA, USA) and adopted the metric cityblock [14] that suggests that the data should be divided in four levels (levels 1 to 4, demarcates the worst to the best performance, respectively). One level was ascribed to each player according to his test scores and the results were recalculated using the means of the cluster analysis. We then performed a sum of these results, considering the different levels achieved by each participant. A new cluster analysis was performed with the data from this summation, which resulted in a general technical ranking.

### 2.4. Statistical Analysis

Statistical analysis was performed using the software IBM^®^ SPSS^®^ Statistics for Windows, version 22.0 (IBM Corporation^©^). Data normality and homogeneity were respectively tested with the use of Shapiro-Wilk and Levene’s tests. The data analysis was obtained by the ranking of the performance obtained from an offensive index of tactical performance and soccer-specific motor skills tests. The Spearman correlation coefficient (rho) was applied to calculate the association between the rankings. In all cases, the significance level was preset at 5% (*p* < 0.05).

## 3. Results

Table 3 presents the descriptive data (mean, standard deviation) of the offensive index of tactical performance and the scores of the soccer-specific motor skills tests that were applied in this study.

Table 4 shows the classification of participants in offensive index of tactical performance and soccer-specific motor skills tests, without the use of cluster analysis (i.e., correlation coefficient of Spearman). There was only a weak positive correlation (rho = 0.39) between the tactical and technical ranking, and it was not significant (*p* = 0.14). This points out that the best classification of the young players of our sample in the technical ranking is not necessarily the same as in the tactical ranking. 

The classification by cluster analysis is described in Table 5. A similar result (rho = 0.35; *p* = 0.20) to the classification without the cluster analysis is observed. The cluster analysis shown in Table 4 demonstrates that participants with an index of tactical performance from 0 to 1.88 were classified Level 1; from 1.89 to 3.52, Level 2; from 3.53 to 4.61, Level 3; and from 4.62 to 5.53, Level 4. In contrast, the sum of the rankings of the motor skills tests’ cluster analysis shows that participants with results from 0 to 9 were classified Level 1; from 10 to 11, Level 2; from 12 to 13, Level 3; and from 14 to 15, Level 4.

## 4. Discussion

This study aims to identify possible associations between tactical knowledge and soccer-specific motor skills with valid scientific protocols. The analysis of the Offensive Index of Tactical Performance provides a specific and contextualized assessment of both the players’ procedural tactical knowledge and their technical skills, while the soccer-specific motor skills tests provide an assessment of technical skills without opposition. Results demonstrated that there is a weak, positive correlation between the offensive procedural tactical knowledge and specific soccer motor skills in young soccer players. We discuss this weak correlation in respect to two general topics: (i) the differences between open and closed tasks in sports, and their implications for the technical assessment of players; (ii) the players’ development.

The scientific literature provides evidence that there are two types of tasks that distinguish the sport disciplines: the “closed tasks”, which are developed in isolated situations to reach a better execution performance [16,17,18,19,20,21], and the “open tasks”, a result of choosing the adequate action to perform according to the situation emerging from the game, and execute it efficiently. This open task is therefore an integration of technique and tactics [5,22,23,24,25,26,27]. Due to the variability and the unpredictability of invasion games, players are constantly required to perceive their environment, anticipate their opponents’ actions and make decisions. These are all tasks that require significant tactical and technical knowledge to solve problems that emerge from an interaction between the players, the tasks (e.g., to get reach the opponents’ goal) and the environment (e.g., the scenario of the game, the conditions of performance) [28]. Therefore, soccer is predominantly characterized by “open tasks” [29,30] despite the existence of isolated “closed tasks” like penalty kicks.

This major difference implies that the assessment tools must adapt and succeed to evaluate the performance of the action in its real context, i.e., with opposition [5,31,32] or in controlled and predetermined contexts [16,17,18,19,20,21,33]. In that sense, many study-based assessments of technical performance in game situations, e.g., small-sided games [34,35], quantify technical actions by counting the number of tackles, dribbles, passes and shots (i.e., notational analysis [36,37,38]).

Due to the variability of the context, it is relevant to outline the following criteria to assess technique [39]: efficient technique, effective technique and adapted technique. An efficient technique refers to the motor action (e.g., a shoot on goal must be executed with the correct foot placement, knee angulations), as defined for a “closed task”. The assessment of an effective technique must consider the result of the action (e.g., did the shoot result in a goal), as defined for “open task”. The adapted technique involves the integration of both the efficiency and the efficacy of the technique in order to meet the demands of results. The unpredictability of the game requires continuous execution of motor gestures according to each game situation. Such performance must therefore be assessed in real game situations, either in small-sided or formal games.

Moreover, the soccer skills tests used in our study [9,10] narrow the players’ full attention on the execution of the motor execution of the assessed movement: this maximizes his internal focus on the movement [7]. In contrast, technical actions in a game context (i.e., small-sided or formal games) require the players to focus on what happens in the game prior to executing any movement, i.e., an external focus [7,28]. This confirms that a successful technical-tactical action is sustained by a decision-making process that results from the exploitation of his tactical and technical knowledge [30,39]. In this sense, we believe that teachers/instructors/researchers involved in soccer should use instruments that prioritize tools allowing the assessment of technical performance in game contexts [7,40].

The tests we used to assess the specific soccer motor skills in our study do assess “closed tasks,” and this may have led to the result of a low correlation between game knowledge and level of specific soccer skills. Praça et al. [7] evaluated 24 young soccer players of 14–15 years of age and also found low correlation between technical and tactical knowledge, thereby corroborating the conclusion presented in our study. We may infer that the motor skills tests’ lack contextual relevance, leaving doubts as to its applicability in assessing representative technical skills for invasion sport games such as soccer [41]. In fact, the motor skills tests do not provide information about the ability to play, the procedural tactical knowledge or the context-specific technical actions of team sports players, whereas performance assessment needs game situation information. The lack of representability of the assessed tasks reveals the inherent needs for the assessment process to focus on the ability to transfer skills from “closed tasks” to “open tasks”, or to adopt different assessment tools.

Recently, Gonçalves et al. [8] showed that technique efficiency increases as the player undergoes physical development and maturation. Malina et al. [42] also showed that the stage of puberty, body size and years of formal training accounted for 21% to 50% of the variance in physical performance of young soccer players (13–15 years). In addition, Malina et al. [43] found that age, experience, body size and stage of puberty contributed significantly in different combinations to the variance in the dribbling, ball control and shooting technical skills tests. On the other hand, Costa et al. [44] also verified the increase of index of tactical performance through age levels. This evidence suggests that the biological maturation and psycho-physical development of children/adolescents influence the performance of technical and tactical actions in soccer during their maturation process. 

Even though our study was realized with a low amount (*n* = 15 players) of young players (10.73 ± 0.46 years) having relatively low training experience (2.53 ± 0.52 years), this research nonetheless contributes to the state of knowledge of the possible relationships between technical and tactical skills. The corroboration with a previous study [7] with 24 federated male soccer players aged 14–15 years (training experience 4.3 ± 1.2 years) also increased the reliability of the featured findings. 

This study also makes the following important points: (i) valid and standardized protocols were used to realize this study; and (ii) the results of our study support the ecological approach to skill acquisition, which suggests that “skill acquisition task protocols should allow performers to use movement variability to explore and create opportunities for action, rather than constraining them to passively receiving information” [45]. 

## 5. Conclusions

Based on these findings, we conclude that there was a weak positive correlation (rho = 0.39, not significant *p* = 0.14—without cluster analysis; rho = 0.35, not significant *p* = 0.20—with cluster analysis) between soccer skills with the ball and offensive tactical knowledge in players of Under-11 age level. The results and discussion presented provide information about the real contribution of assessment of the performance invasion games such as soccer. We show how important it is to reflect on the use of tests for technical skills without a game context since they do not consider the unpredictability and variability of the game. For example, freestyle soccer players certainly get a good ranking in the ball control test, but it does not guarantee that these players will perform well in the soccer match.

## Figures and Tables

**Table 1:** Sample characterization.

Variables	Mean ± Standard Deviation
Age (years)	10.73 ± 0.46
Experience level (years)	2.53 ± 0.52
Height (cm)	150.47 ± 8.13
Body Mass (kg)	44.79 ± 11.64
Peak Height Velocity (years)	−1.79 ± 0.63
% of fat	20.49 ± 7.63

**Table 2:** Ranking of soccer specific motor skills tests.

Participants	Ranking Ball Control	Ranking Shooting	Ranking Passing	Ranking Dribbling	Sum of Ranking	Final Ranking
1	4°	2°	2°	14°	22°	4.5°
2	1°	14°	2°	6°	23°	6°
3	3°	7°	5°	7°	22°	4.5°
4	15°	9°	11°	11°	46°	14°
5	2°	5°	1°	8°	16°	2°
6	4°	2°	5°	2°	13°	1°
7	11°	9°	9°	13°	42°	13°
8	7°	5°	8°	5°	25°	7°
9	12°	14°	11°	4°	41°	12°
10	8°	1°	2°	9°	20°	3°
11	14°	9°	14°	15°	52°	15°
12	8°	9°	9°	1°	27°	8°
13	12°	7°	15°	3°	37°	11°
14	10°	9°	5°	10°	34°	10°
15	6°	4°	11°	12°	33°	9°

**Table 3:** Offensive index of tactical performance descriptive data and specific soccer skills tests results (ball control, shooting, passing and dribbling).

Variables	Mean	Standard Deviation
Offensive Index of Tactical Performance (AU)	4.11	1.03
Ball Control (touches)	26.13	39.97
Shooting (points)	5.73	3.03
Passing (number of passes)	12.80	2.65
Dribbling (seconds)	15.12	1.48

**Table 4:** Association between technical ranking and tactical ranking without cluster analysis *.

Participants	Offensive Index of Tactical Performance	Tactical Ranking	Sum of the Ranking of the Specific Soccer Skills Tests	Technical Ranking	rho Value	*p* Value
1	5.53	1°	22°	4.5°	0.39	0.14
2	5.38	2°	23°	6°		
3	5.33	3°	22°	4.5°		
4	5.13	4°	46°	14°		
5	4.61	5°	16°	2°		
6	4.39	6°	13°	1°		
7	4.29	7°	42°	13°		
8	4.18	8°	25°	7°		
9	4.00	9.5°	41°	12°		
10	4.00	9.5°	20°	3°		
11	3.52	11°	52°	15°		
12	3.43	12°	27°	8°		
13	3.00	13°	37°	11°		
14	2.91	14°	34°	10°		
15	1.88	15°	33°	9°		

* rho = Correlation coefficient; *p* = significance.

**Table 5:** Association between technical ranking and tactical ranking with cluster analysis *.

Participants	Offensive Index of Tactical Performance	Tactical Ranking	Sum of the Ranking of the Specific Soccer Skills Tests	Technical Ranking	rho Value	*p* Value
1	5.53	4°	12°	3°	0.35	0.20
2	5.38	4°	12°	3°		
3	5.33	4°	14°	4°		
4	5.13	4°	7°	1°		
5	4.61	3°	15°	4°		
6	4.39	3°	15°	4°		
7	4.29	3°	9°	1°		
8	4.18	3°	14°	4°		
9	4.00	3°	7°	1°		
10	4.00	3°	12°	3°		
11	3.52	2°	6°	1°		
12	3.43	2°	11°	2°		
13	3.00	2°	8°	1°		
14	2.91	2°	10°	2°		
15	1.88	1°	11°	2°		

* rho = Correlation coefficient; *p* = significance.
